# Bi-Magnetic Core-Shell CoFe_2_O_4_@MnFe_2_O_4_ Nanoparticles for In Vivo Theranostics

**DOI:** 10.3390/nano10050907

**Published:** 2020-05-08

**Authors:** Valentin Nica, Carlos Caro, Jose Maria Páez-Muñoz, Manuel Pernia Leal, Maria Luisa Garcia-Martin

**Affiliations:** 1 BIONAND—Centro Andaluz de Nanomedicina y Biotecnología (Junta de Andalucía, Universidad de Málaga), Parque Tecnológico de Andalucía, 29590 Málaga, Spain; nicaval@gmail.com (V.N.); ccaro@bionand.es (C.C.); jpaez@bionand.es (J.M.P.-M.); 2 Department of Physics, “Alexandru Ioan Cuza” University of Iasi, 700506 Iasi, Romania; 3 Departamento de Química Orgánica y Farmacéutica, Facultad de Farmacia, Universidad de Sevilla, 41012 Seville, Spain; 4 Networking Research Center on Bioengineering, Biomaterials and Nanomedicine, CIBER-BBN, 29590 Malaga, Spain

**Keywords:** bi-magnetic nanoparticles, contrast agents, MRI, SAR, theranostics

## Abstract

In this work, we report the synthesis and characterization of three magnetic nanosystems, CoFe_2_O_4_, CoFe_2_O_4_@ZnFe_2_O_4_, and CoFe_2_O_4_@MnFe_2_O_4_, which were developed as potential theranostic agents for magnetic hyperthermia and magnetic resonance imaging (MRI). These nanosystems have been thoroughly characterized by X-ray Diffraction (XRD), Transmission Electron Miscroscopy (TEM), Dark Field-TEM (DF-TEM), Vibrating Sample Magnetometry (VSM), and inductive heating, in order to elucidate their structure, morphology, and magnetic properties. The bi-magnetic CoFe_2_O_4_@ZnFe_2_O_4_ and CoFe_2_O_4_@MnFe_2_O_4_ nanoparticles (NPs) exhibited a core-shell structure with a mean average particle size of 11.2 ± 1.4 nm and 14.4 ± 2.4 nm, respectively. The CoFe_2_O_4_@MnFe_2_O_4_ NPs showed the highest specific absorption rate (SAR) values (210–320 W/g) upon exposure to an external magnetic field, along with the highest saturation magnetization (Ms). Therefore, they were selected for functionalization with the PEGylated ligand to make them stable in aqueous media. After the functionalization process, the NPs showed high magnetic relaxivity values and very low cytotoxicity, demonstrating that CoFe_2_O_4_@MnFe_2_O_4_ is a good candidate for in vivo applications. Finally, in vivo MRI experiments showed that PEGylated CoFe_2_O_4_@MnFe_2_O_4_ NPs produce high *T*_2_ contrast and exhibit very good stealth properties, leading to the efficient evasion of the mononuclear phagocyte system. Thus, these bi-magnetic core-shell NPs show great potential as theranostic agents for in vivo applications, combining magnetic hyperthermia capabilities with high MRI contrast.

## 1. Introduction

Nowadays, cancer continues to be one of the most frequent and severe diseases, with a global estimate in 2018 of 18.1 million new cases and 9.6 million deaths [1]. However, in recent years, there has been an improvement in the survival rate of cancer patients thanks to preventive activities, early diagnosis campaigns, and therapeutic advances [2]. In vivo imaging techniques, such as Magnetic Resonance Imaging (MRI), Computed Tomography (CT), or Positron Emission Tomography (PET), among others, have played a fundamental role in early diagnosis. MRI is unquestionably the most versatile of all of them, being able to provide both morphological and functional information with excellent image quality, especially from soft tissues. In addition, MRI has the advantage of using non-ionizing radiation and, in most cases, it does not need any exogenous substance to obtain good tissue contrast [3]. Nevertheless, MRI contrast agents (CA) are also frequently used as a source of extrinsic contrast to better visualize an area of interest or to obtain dynamic information, such as blood flow, blood volume, etc. [4,5]. The most commonly used CAs for MRI are the Gadolinium complexes. However, Gd-based CAs can produce toxic effects, not only in patients with renal impairment, as firstly described, but also in patients with normal renal function, as demonstrated by subsequent studies, in which the accumulation of Gd was observed in different tissues, including brain, kidneys, and bone [6,7]. In the last few years, there has been a growing interest in the development of new CAs based on nanotechnology in order to overcome this limitation [8,9,10,11]. In this sense, bi-magnetic core-shell nanoparticles (NPs) have emerged as a very promising option for the development of new MRI CAs with outstanding magnetic properties [12,13,14]. Fe@MFe_2_O_4_ (M=Fe, Mn, Co) core-shell NPs demonstrate higher transverse relaxivity (*r*_2_) than their single-core counterpart with similar size [15]. Additionally, bi-magnetic NPs can also be used for drug delivery [16] and magnetic hyperthermia [17], thus being excellent candidates for the development of novel nano-theranostic agents. The use of magnetic hyperthermia for tumor therapy is based on the fact that cancer cells are more sensitive to small increases in temperature than healthy cells. Therefore, an increase in local temperature that is mediated by magnetic NPs accumulated in the tumor can kill cancer cells with a minimal effect on healthy tissue [18,19]. Magnetic hyperthermia can be defined as the generation of heat upon the exposure of magnetic NPs to high-frequency alternating magnetic fields (HF-AMF) [20,21,22]. The heating capability of bi-magnetic NPs can be evaluated through their specific absorption rate (SAR), which is related to surface and shape anisotropy, thee degree of spin disordering, and exchange anisotropy at the interface between the magnetic phases (exchange-coupling phenomenon). Importantly, the bi-magnetic core-shell structure has been shown to increase their heating efficiency by an order of magnitude when compared to its single-core counterpart [23]. In addition, the heating capability of magnetic NPs is dependent on the chemical composition and microstructural features, which, to some extent, can be controlled by the synthesis route [24,25]. Thus, Khurshid et al. showed that both SAR and heating efficiency of the FeO/Fe_3_O_4_ core-shell architecture are improved by tuning the effective anisotropy of the nanosystem [26]. In addition, bi-magnetic NPs that consist of both soft and hard magnetic phases can greatly enhance their SAR due to the interfacial exchange coupling between the magnetic core-shell layers [23,27]. CoFe_2_O_4_ has been extensively explored as a hard magnetic material with high coercivity (H_c_) and high saturation magnetization (M_S_) [28,29], whereas MnFe_2_O_4_ possesses soft magnetic properties, with low H_c_ and moderate M_S_ [30,31]. Various studies have shown that the magnetic properties of CoFe_2_O_4_@MnFe_2_O_4_ and MnFe_2_O_4_@CoFe_2_O_4_ NPs can be controlled through the variation of their magnetic hard/soft volume ratio [32,33]. Thus, the versatility of their properties can be used to design efficient theranostic platforms [34]. However, bi-magnetic NPs also have some drawbacks, such as their low colloidal stability, which might lead to limitations for in vivo applications. The magnetic particle surface can be functionalized with various polymers, such as dextran, polyvinyil alcohol (PVA), polyvinylpyrrolidone (PVP), or polyethylene glycol (PEG), to overcome this problem [35]. PEG has been widely used because of its solubility in polar and non-polar solvents, biocompatibility, and anti-fouling properties. The functionalization of nanoparticle surfaces with PEG has been demonstrated to reduce cytotoxicity and prolong blood circulation time in vivo [36].

Herein, three magnetic nanosystems, comprising single core (CoFe_2_O_4_) and core-shell NPs (CoFe_2_O_4_@MnFe_2_O_4_ and CoFe_2_O_4_@ZnFe_2_O_4_), are investigated as potential multifunctional nanosystems for both MRI and magnetic hyperthermia. We were able to reduce the thickness of the shell and, therefore, to decrease the hard/soft volume ratio by tuning the synthesis conditions of bi-magnetic NPs. This led to a stronger interfacial exchange coupling between magnetic layers and, consequently, to an increase in SAR, while avoiding an excessive increase in the particle size. Subsequently, the nanosystem with the highest SAR and Ms values was functionalized with a PEGylated ligand to render water-soluble bi-magnetic NPs with high colloidal stability. The relaxivity, biodistribution, and pharmacokinetics studies demonstrated the high potential of these bi-magnetic NPs for theranostic applications.

## 2. Experimental Section

### 2.1. Materials

The chemicals and solvents were obtained from commercial suppliers (Sigma Aldrich, Acros Organics and Fisher Scientific, St. Louis, MO, USA) and were used as received. Iron (III) acetylacetonate (Fe(acac)_3_), Cobalt (II) acetylacteonate (Co(acac)_2_), Manganese (II) acetylacteonate (Mn(acac)_2_), Zinc (II) acetylacteonate Zn(acac)_2_, oleylamine, oleic acid, 1,2-hexadecanediol, Gallic acid, Poly ethylene glycol 3000 Da,1-octadecene, diphenylether, benzyl ether, Triethylamine, 4-Dimethylaminopyridine, dicyclohexyl carbodiimide (DCC), Hydrochloric acid (HCl), Sodium sulfate (Na_2_SO_4_), 3-[4,5-dimethylthiazol-2yl]-2,5-diphenyl tetrazolium bromide (MTT), Phosphate Buffered Saline (PBS), and Dulbecco’s Modified Eagle’s Medium (DMEM). As solvents, Milli-*Q* water (18.2 MΩ, filtered with filter pore size 0.22 µM) from Millipore, toluene, ethanol, acetone, dimethylsulphoxide (DMSO), hexane, chloroform, dichloromethane, and tetrahydrofuran were used anhydrous and HPLC grade.

### 2.2. Synthesis of the Nanoparticles

- Synthesis of single-core nanoparticles.

The singleCoFe_2_O_4_ (CF) NPs were prepared by mixing 1 mmol of Co(acac)_2_, and 2 mmol of Fe(acac)_3_ with 10 mmol 1,2-hexadecandiol, 6 mmol of oleic acid, 6 mmol of oleylamine in 20 mL benzyl ether. The solution was heated at 100 °C for 30 min. under nitrogen atmosphere to remove the air and moisture. The reaction mixture was heated at 200°C for 2 h, then at 290 °C for 1 h, and subsequently cooled down to room temperature. The particles were washed with ethanol and hexane (1:1) three times before sedimentation. Finally, the NPs were re-suspended in hexane.

- Synthesis of core-shell bi-magnetic nanoparticles.

The core-shell CoFe_2_O_4_@MnFe_2_O_4_ (CF@MF) NPs were synthesized by the seed-mediated growth method. 80 mg of CF particles suspended in hexane were added to a reaction system that was similar to that described above containing 1 mmol Mn(acac)_2_. The mixture was heated slowly for 30 min. to 100 °C, and then heated to 300 °C (a heating rate of 8 °C/min.) for 30 min. under nitrogen atmosphere. Subsequently, ethanol was added to the mixture after cooling, resulting in a black precipitate. The CF@MF NPs were dispersed in hexane after centrifugation. Similarly, CoFe_2_O_4_@ZnFe_2_O_4_ (CF@ZF) NPs were synthesized by the seed-mediated growth method. 80 mg of CF particles suspended in hexane were added to a reaction system similar to that described above containing 1 mmol Zn(acac)_2_. The mixture was heated slowly for 30 min. to 100 °C and then heated to 300 °C (heating rate of 8 °C/min.) for 30 min. under nitrogen atmosphere. Subsequently, ethanol was added to the mixture after cooling, resulting in black precipitate. The CF@ZF NPs were dispersed in hexane after centrifugation.

### 2.3. Synthesis of the PEGylated Ligand

**Figure 1:**
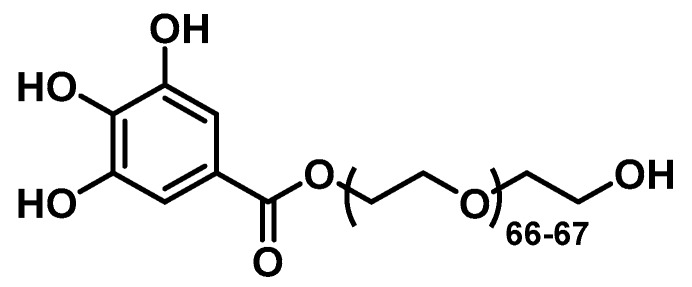
Chemical structure of the gallol-PEG66-67-OH ligand.

The gallol-PEG_66-67_-OH (Figure 1) was synthesized following the synthetic route that was previously reported by us [9,10,11]. Briefly, to a solution of poly ethylene glycol (Mw: 3000 g/mol, 1 mmol, 3.0 g), gallic acid (Mw: 170 g/mol, 1 mmol, 170 mg), and 4-(dimethylamino) pyridine (Mw: 122 g/mol, 200 μmol, 24 mg) in 100 mL of tetrahydrofuran and 10 mL of dichloromethane, in a round-bottom flask under nitrogen atmosphere, was added dropwise to a solution of dicyclohexyl carbodiimide (Mw: 206 g/mol, 5 mmol, 1 g). The mixture was stirred overnight at room temperature. The reaction mixture was filtered through a filter paper and the solvents were rota-evaporated. ^1^H NMR spectroscopy confirmed the obtaining of the desired product gallol-PEG_66-67_-OH. ^1^H NMR (400 MHz, CDCl_3_) *δ* (ppm): 7.22 (s, 2H), 4.43-4.40 (m, 2H), and 3.85-3.45 (m, CH_2_-PEG, -OH). FTIR peaks (cm^−1^): 1466 (C-H bend vibration), 1359 (C-H bend vibration), 1341 (C-H bend vibration), 1307 (anti-symmetric stretch vibration), 1268 (C-O stretch vibration), 1238 (C-O stretch vibration), 1092 (C-O-C stretch vibration), and 942 (CH out-of-plane bending vibration).

### 2.4. Functionalization of Bi-Magnetic NPs

The functionalization of the CF@MF was performed following a previously published protocol [37]. Briefly, a solution of 1.0 mL of CF@MF NPs (10 g/L of Fe), 1.0 mL of the derived gallol-PEG_66-67_-OH at a concentration of 0.1 M in CHCl_3_ and 50 µL of triethylamine were added in a separating funnel. The mixture was gently shaken and diluted with 5 mL of toluene, 5 mL of milli-*Q* water, and 10 mL of acetone. Subsequently, it was shaken again and the NPs were transferred into the aqueous phase. The aqueous phase was collected in a round-bottom flask and the residual organic solvents were rota-evaporated. After that, the gallol derived NPs were purified while using centrifuge filters with a molecular cut-off weight of 100 kDa at 450 rcf. In each centrifugation, the functionalized NPs were re-suspended in milli-*Q* water. The purification step was repeated several times until the filtered solution was clear. Afterwards, the gallol derived NPs were re-suspended in PBS buffer. Finally, the solution of NPs was centrifuged at 150 rcf for 5 min. and also placed onto a permanent magnet (0.6 T) for 5 min. to ensure that highly stable mono-dispersed magnetic NPs are obtained.

### 2.5. Characterization Methods

- *Transmission Electron Microscopy*.

The TEM images were obtained on a FEI Tecnai G2 Twin microscope (FEI, Hillsboro, OR, USA) operated at an accelerating voltage of 100 kV. TEM samples were prepared by dropping a solution of the corresponding NPs at ~1 g/L of Fe on a carbon-coated copper grid and letting the solvent evaporate. The mean diameter size was calculated from the bright-field TEM micrographs, on an average of hundred NPs measured.

- *X-ray diffraction patterns (XRD)*.

The powder X-ray diffraction patterns were measured using a PANanalyticalX’PertPro diffractometer (PANalytical B.V., Almelo, The Netherlands) using Cu_K*α*_ radiation (*λ* = 1.54059 Å). The samples were analyzed in the range of 2*θ* = 20° to 80°, with scanning angle rate of 0.02° and 2 s/step count time.

- *Vibrating Sample Magnetometer (VSM)*.

The magnetization curves were studied by means of a vibrating sample magnetometer (MicroMag 3900, Princeton Measurements Corp., Montgomery, NJ, USA) in external magnetic fields from −1 T to 1 T.

- *Specific Absorption Rate (SAR)*.

Hüttinger Elektronik (IG5) (Hütinger Elektronik GmbH & Co. KG, Freiburg, Germany), which supplies a high frequency applying magnetic field (1950 kHz) with a maximum power of 7.2 kW, was utilized to perform SAR measurements of the Core-Shell NPs in the medium. The Core-Shell NPs samples (1 mg/mL) were placed in a water-cooled magnetic induction coil. The temperature variations over time were registered using a fiber optic sensor (OPT OCON AG) (Optocon AG, Dresden, Germany).

The SAR values of the NPs in aqueous medium were calculated from the time dependence of the temperature on the radiofrequency electromagnetic field (1950 kHz), according to the equation:

$$SAR=\frac{c}{m}\times \frac{dT}{dt}$$

where *c* is the specific heat capacity of the medium and *m* is the mass of the sample.

- *Dynamic Light Scattering (DLS)*.

The size distribution and Zeta potential measurements of the gallol-derived magnetic NPs were performed on a Zetasizer Nano ZS90 (Malvern Instruments, Malvern, Worcestershire, UK). The NPs were dispersed in milli-*Q* water or PBS at a concentration of 50 mg/L of Fe content. The measurements were completed on a cell type: ZEN0118-low volume disposable sizing cuvette, setting 2.420 as refractive index with 173° Backscatter (NIBS default) as angle of detection. The measurement time was set to automatic and three measurements were performed for each sample. As an analysis model, the general purpose (normal resolution) was chosen. The number mean was selected for the size distribution measurement.

- *Nuclear Magnetic Resonance Spectroscopy (NMR)*.

The ^1^HNMR spectra of the PEGylated ligandprepared in CDCl_3_ were recorded on a NMR Bruker Ascend 400MHz spectrometer (Bruker BioSpin, Rheinstetten, Germany).

- *Inductively Coupled Plasma High Resolution Mass Spectroscopy (ICP-HRMS)*.

The Fe, Co, Zn, and Mn concentrations were determined on an ICP-HRMS. Magnetic NPs were digested with aqua regia (a mixture of three parts of HNO_3_ and one part of HCl). Briefly, 2.5 mL of aqua regia were added to 25 μL of a solution of NPs in a volumetric flask. The mixture was left overnight. Subsequently, milli-*Q* water was added to complete a total volume of 25 mL.

- *In vitro longitudinal and transversal relaxivities (r_1_ and r_2_)*.

Magnetic relaxivities (*r*_1_ and *r*_2_) were calculated in PBS at low magnetic field, 1.44 T, and at high magnetic field, 9.4 T, using concentrations of gallol-derived NPs between 2 to and 0.2 mM of Fe, at 37 °C. Low magnetic field measurements were performed on a Bruker Minispec system (Bruker BioSpin, Rheinstetten, Germany. *T*_1_ was determined using an inversion-recovery sequence, and *T*_2_ using the Carl-Purcell-Meiboom-Gill (CPMG) spectroscopy sequence. High magnetic field (9.4 T) measurements were performed on a Bruker Biospec MRI system (Bruker BioSpin, Ettlingen, Germany) equipped with 400 mT m^−1^ field gradients and a 40 mm quadrature bird-cage resonator. *T*_1_ values were determined with a saturation recovery image sequence, and *T*_2_ values with a 64-echo Carl-Purcell-Meiboom-Gill (CPMG) sequence (TE values from 7.5 ms to 640 ms). 

The relaxivities, *r*_1_ and *r*_2_, at both magnetic fields were calculated from the slope of the linear regression of the relaxation rate (1/*T*_1_ or 1/*T*_2_) versus the Fe concentration.

- *Cell Culture*.

The C6 rat glioma cells were cultured in Dulbecco’s Modified Eagle Medium (DMEM) supplemented with 2 mM *L*-glutamine, 10% fetal bovine serum (FBS) and 1% penicillin/streptomycin, at 37 °C in an incubator with a humidified atmosphere containing 5% CO_2_.

- *Cytotoxicity assays*.

Cytotoxicity was evaluated in C6 cells using the MTT assay, which is described in detail in the supporting information.

- *In vivo Magnetic Resonance Imaging*.

The in vivo mice experiments were performed in accordance with the ethical guidelines of our local ethical committee and consistent with national regulations for the care and use of laboratory animals (R.D. 53/2013). Male Balb/c mice (*n* = 3) with ca. 22 g in weight, provided by Janvier Labs (Le Genest-Saint-Isle, France) were used. The animals were anesthetized with 1% isoflurane, the tail vein was cannulated and then the animals were placed in the magnet, where respiration and body temperature were monitored throughout the entire MRI experiment. The magnetic NPs were intravenously administered via tail vein at a concentration of 10 mg of Fe per kg of body weight.

All of the MRI experiments were carried out on the 9.4 T Bruker system (Bruker Biospec, Bruker BioSpin, Ettlingen, Germany) described above. High resolution *T*_2_-weighted images were acquired using a turbo-RARE sequence with respiratory gating (TE = 16 ms, TR = 1000 ms, 4 averages, 156 μm in-plane resolution, and 1 mm slice thickness). Quantitative *T*_2_ measurements were also performed using a multi-echo spin echo sequence (TEs ranging from 7 ms to 448 ms, TR = 3500 ms, FOV = 4 cm, matrix size = 128 × 128, slice thickness = 1 mm). The time-courses were followed by using a turbo-RARE sequence with the same parameters indicated above, but only one average was used to improve the temporal resolution (one image every 30 s). The acquisition scheme was as follows: *T*_2_-weighted, quantitative *T*_2_, intravenous injection of the gallol-derived magnetic NPs, time-course for 35 min., quantitative *T*_2_ and *T*_2_ weighted. The first 35 min. time courses were semi-quantitatively analyzed while using the following expression:

$$RE=\left|\frac{I_{t}-I_{0}}{I_{0}}\times 100\right|$$

where *RE* is the modulus of relative signal enhancement, *I_t_* is the signal intensity at any given time after the NPs injection, and *I*_0_ is the signal intensity before the injection. 

Long-term pharmacokinetics were measured by quantitative *T*_2_ mapping at 0 h, 1 h, and 24 h. Pharmacokinetics were obtained by calculating the average values within different regions of interest (ROIs) placed on the following tissues: liver, kidneys, and muscle.

- *Histology*.

After 24 h of NPs intravenous administration, the mice were sacrificed and the kidney and liver extracted. Haematoxylin and Eosin (H&E) staining was used to assess tissue architecture, and Prussian blue staining to visualize iron deposits. The detailed procedures are described in the supporting information.

### 2.6. Statistical Analysis

Statistical analysis was performed while using the SPSS package (v20, SPSS Inc., Chicago, IL, USA). Cell viability and in vivo *T*_2_ values are shown as mean ± standard deviation (SD). Student’s *t*-test or one-way analysis of variance (ANOVA) was used to determine the significant differences between different NPs or different experimental conditions. The level of significance was set at *p* < 0.05.

## 3. Results and Discussion

The seed-mediated growth method had been demonstrated to be an efficient way to synthesize uniform and monodisperse bi-magnetic NPs [38].Thus, two different bi-magnetic CoFe_2_O_4_@MnFe_2_O_4_ and CoFe_2_O_4_@ZnFe_2_O_4_NPs were synthesized by this method while using CoFe_2_O_4_ (CF) as seed, and tuning the precursors ratio and the temperature ramp to obtain the desired core-shell NPs. These new bi-magnetic NPs were thoroughly characterized by different physicochemical techniques, as described below.

### 3.1. X-ray Diffraction Analysis

**Figure 2:**
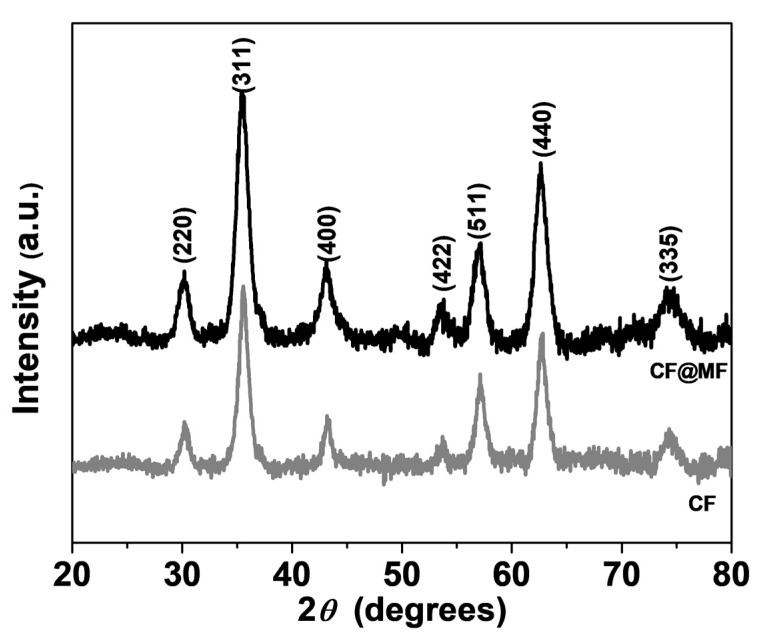
X-ray diffraction (XRD) patterns of CoFe_2_O_4_ (CF)(grey) and CoFe_2_O_4_@MnFe_2_O_4_ (CF@MF) (black) nanoparticles (NPs).

Figure 2 and Figure S1 show the X-ray diffraction patterns, where the Bragg reflections of all samples match the reported values for the cubic structure with the space group Fd-3m (JCPDS, card no 22-1086). No impurity phases were observed.

The mean crystallite size <*d*>, lattice parameter (a) and microstrain (*ε*) (Table 1 and Table S1) were calculated for each sample from the diffraction pattern by Rietveld refinement implemented in TOPAS Academic v4.1 software (v4.1, Coelho Software, Brisbane, Australia).

The mean crystallite size of the single particle samples was calculated to be 9.8 nm, whereas, for the bi-magnetic NPs, the <*d*> values were slightly increased (10.4 nm for CF@MF). The lattice parameters of all the samples did not present significant deviations from the standard value (a = 8.388 Å). The low microstrain values demonstrated that the NPs were not affected by lattice defects [39].

### 3.2. Transmission Electron Microscopy

**Figure 3:**
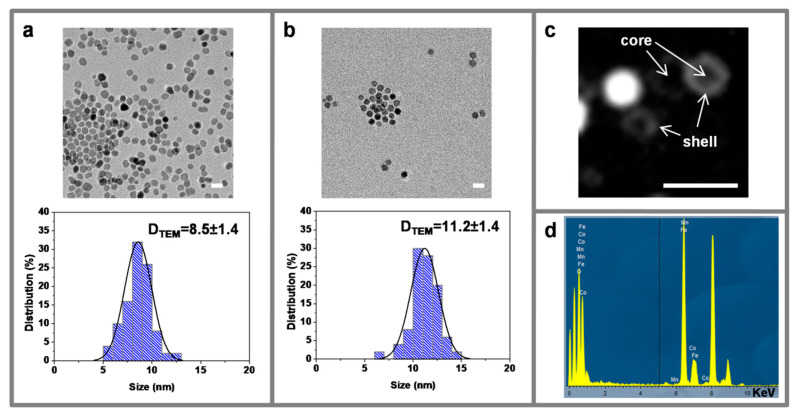
Representative Transmission Electron Miscroscopy (TEM) images (top) of CF (**a**) and CF@MF (**b**) NPs, and their corresponding size distribution histograms (bottom). TEM diameters are expressed as the mean ± SD by measuring at least 100 particles. Representative Dark field-TEM images of CF@MF NPs (**c**). Scale bar is equivalent to 20 nm in all images. EDX spectrum of CF@MF nanoparticles (**d**).

All of the NPs showed monodisperse spherical shapes with different diameters, depending on the composition, being 8.5 ± 1.2 nm for the CF (Figure 3a), 11.2 ± 1.4 nm for the CF@MF (Figure 3b), and 14.4 ± 2.4 nm for the CF@ZF (Figure S2). Dark field transmission electron microscopy (DF-TEM) can be used to discern different compositions based on contrast variations, since this technique predominantly uses Rutherford scattered electrons, which present greater susceptibility to small differences in atomic number (*Z*) [40]. Therefore, the morphology of the core-shell NPs was studied by DF-TEM, as previously reported [17,41]. The results confirmed the hard/soft magnetic phases of the CF@MF NPs (Figure 3c). In addition, the manganese composition was confirmed by EDX, showing the typical peaks that were associated to it (Figure 3d).

### 3.3. Magnetic Characterization

**Figure 4:**
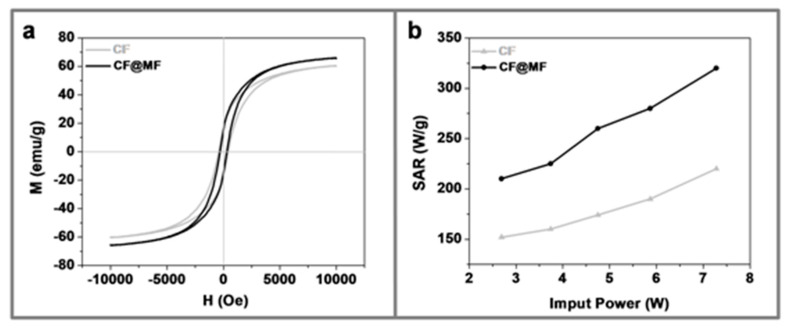
(**a**) Magnetization curves of CF (grey) and CF@MF (black) nanoparticles. (**b**) SAR for different power values of the CF (grey) and CF@MF (black) nanoparticles (at a frequency of 1950 kHz).

Figure 4a and Figure S2a show the specific saturation magnetization (Ms) and the coercivity (Hc) of CF, CF@ZF, and CF@MF. Figure 3a and Figure S2a show the hysteresis loops of the powders determined at room temperature. Ms values were 65.6 emu/g for CF@ZF and 64.2 emu/g for CF@MF, with Hc of 298.3 Oe for both CF@MF and CF@ZF. The CF NPs presented lower Ms value (60.7 emu/g) and higher Hc value (398.2 Oe) when compared to the bi-magnetic samples. The findings reported in this work, showing that Ms values are affected by the diameter of the NPs, being higher for larger diameters, are in good agreement with previous studies [42]. In addition, the Ms values reported therein (54.7 emu/g for 9.6 nm NPs) are very similar to ours, although, in our case, it cannot be ruled out that the different metal composition of NPs also had an effect on this parameter. Lastly, the hysteresis loops confirmed the superparamagnetic behavior of our NPs.

The SAR values for CF@ZF were between 180 and 290 W/g (Figure S3b), whereas, in the case of CF@MF, the SAR values ranged between 210–320 W/g, with these values being the highest among all the NPs tested (Figure 4b). The SAR values of the bi-magnetic NPs were higher than those of the CF (152–220 W/g) and similar to the SAR values of other types of core-shell NPs [43,44]. 

The increase of both Ms and SAR of the hard/soft core-shell NPs when compared to their hard core counterpart can be related to the exchange-coupling phenomenon at the hard-soft interface [23,34,44].

The hysteresis loops measurements could also confirm the magnetic coupling, where we observed an increase in Ms of both CF@ZF and CF@MF core-shell NPs, as compared to the hard core component. 

In summary, these results show that CF@MF NPs show the best combination of Ms and SAR values and, therefore, they were selected for further experiments.

### 3.4. Fourier-Transform Infrared Spectroscopy(FTIR) 

The CF@MF NPs were functionalized with a gallol-derived PEG ligand to make them biocompatible, aiming at biomedical applications. The presence of the gallol-derived ligand that was attached to the surface of the nanoparticle was analyzed and confirmed by FTIR spectroscopy (Figure S4).

### 3.5. Dynamic Light Scattering (DLS)

**Figure 5:**
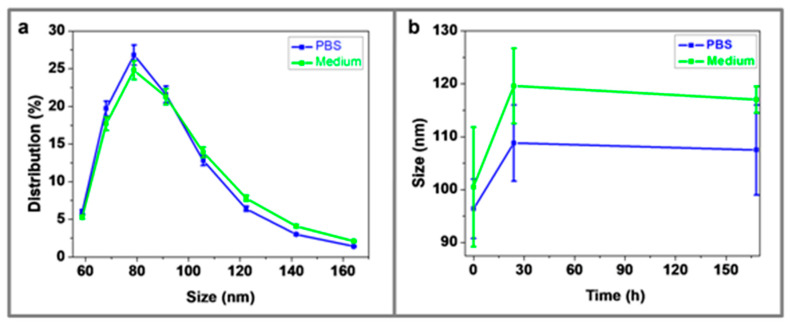
(**a**) Histogram of Dynamic Light Scattering (DLS) sizes in Phosphate Buffered Saline (PBS) (blue) and cell growth medium (green); (**b**) hydrodynamic diameters of magnetic NPs vs time at 37 °C in PBS buffer (blue) and cell growth medium (green).

The hydrodynamic diameter (HD) of PEGylated CF@MF NPs determined by DSL was around 90 nm, both in PBS and in cell growth medium (Figure 5a). A moderate increase of the HD (~20%) could be observed during the first 24 h and it then remained stable over one week (Figure 5b).Thus, it can be concluded that the functionalization of NPs by PEGylation endows high stability in physiological media, which is a key requirement for biological applications.

### 3.6. Magnetic Relaxivity

**Figure 6:**
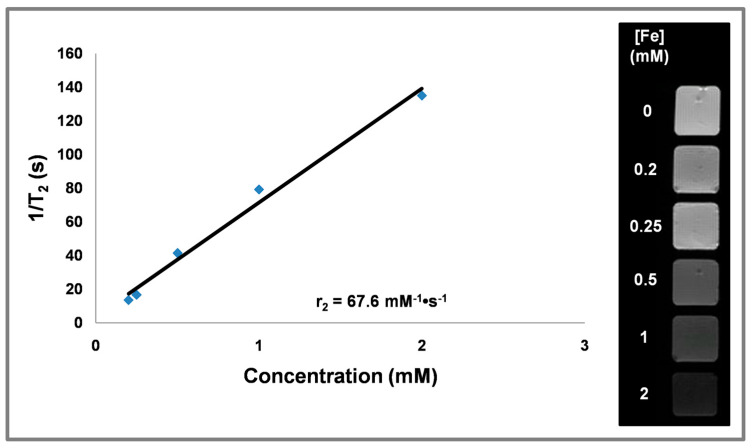
Plot of transverse relaxation rate (1/*T*_2_) over the Fe concentration of PEGylated CF@MF NPs, measured at 9.4 T (left). Transverse relaxivity (*r*_2_) corresponds to the slope of the regression (black line). Representative *T*_2_-weighted MRI image of a phantom containing solutions of PEGylated CF@MF NPs at different concentrations (right).

The transversal relaxivity (*r*_2_) of CF@MF was 30.2 mM^−1^·s^−1^at 1.5 T and 67.6 mM^−1^·s^−1^ at 9.4 T (Figure 6 and Figure S5). Regarding *T*_1_ relaxivity (*r*_1_), CF@MF showed very low values, 1.4 mM^−1^·s^−1^ at 1.5 T (Figure S6), indicating that these NPs behave as *T*_2_ contrast agents, with *r*_2_/*r*_1_ = 21.5. Similarly, at 9.4 T, PEGylated CF@MF NPs showed an *r*_1_ value close to 0 (data not shown).

### 3.7. Cytotoxicity Assays 

Cytotoxicity was evaluated in C6 rat glioma cells. The cultured cells were exposed to increasing concentration of magnetic NPs, from 0.1 μg/mL to 100 μg/mL Fe. After 24 h, the assay showed more than 80% of normal mitochondrial activity, even at the high concentration of 50 μg/mL (*p* < 0.05). These results support the potential use of these NPs for biomedical applications. 

### 3.8. In Vivo MRI Studies

We further proceed with the in vivo MRI studies based on the experimental results described above, showing the high stability in physiological media and the low cytotoxicity of the CF@MF NPs.

**Figure 7:**
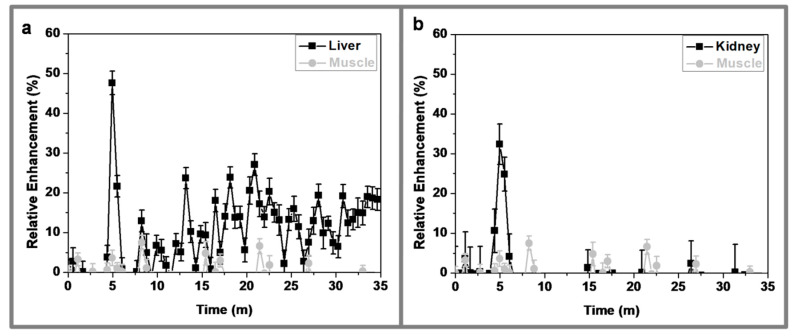
In vivo time courses of PEGylated CF@MF nanoparticles after intravenous injection in balb/c mice: (**a**) liver (black) and muscle (grey), (**b**) kidneys (black) and muscle (grey).

The short term pharmacokinetics, followed by dynamic MRI, showed a rapid increment (around 50%) of the relative enhancement (RE) in the liver, followed by a quick decrease to basal levels and a second phase of slow increment, reaching around 20% after 30 min. of intravenous injection (Figure 7). This behavior indicates that, after injection, the NPs pass through the liver, but they are not immediately retained within this organ; on the contrary, they continue circulating until they are slowly taken up by the liver in a second clearance phase. This slow clearance implies that PEGylated CF@MF NPs present very good stealth properties and, therefore, avoid rapid recognition by the mononuclear phagocyte system (MPS), which leads to longer circulation times and higher bioavailability. In the case of the kidneys, they also showed a rapid increase in the RE curve (around 40%) that, similarly to the liver, rapidly returned to the basal levels, where it remained during the rest of the dynamic study (30 min.). This means that PEGylated CF@MF NPs were not retained in the kidneys, in disagreement with previous reports showing retention in the renal cortex of NPs with HDs ranging from 76 to 96 nm. These results support the fact that nanoparticle size is not the only parameter determining renal retention or liver uptake.

**Figure 8:**
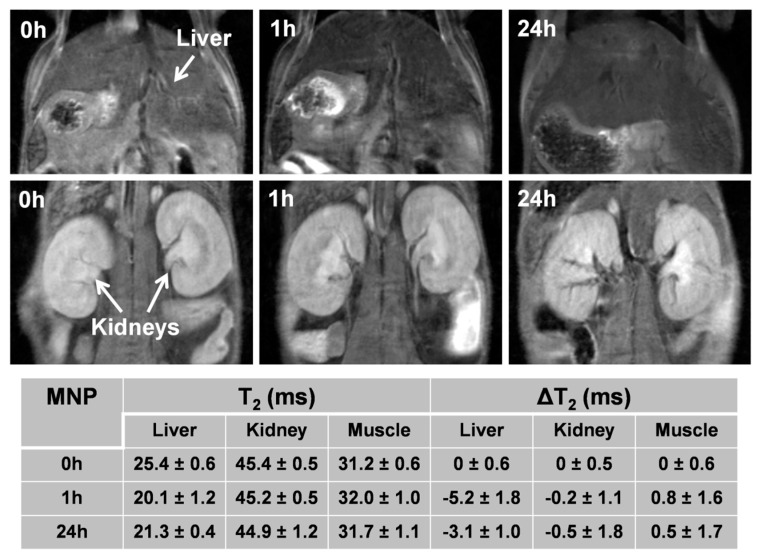
Representative*T*_2_-weighted MR images at different experimental times after the intravenous injection of PEGylated CF@MFNPs of liver (top) and kidneys (bottom). *T*_2_ and Δ*T*_2_ values of liver, kidneys and muscle at different times after the intravenous injection of PEGylated CF@MF NPs. Values correspond to the mean ± standard deviation (*n* = 3).

Long term pharmacokinetics were evaluated by quantitative *T*_2_ mapping at 0, 1h, and 24 h, and high resolution *T*_2_-weighted images were also acquired at each time point (Figure 8). At 1 h post administration, the liver showed a decrease in *T*_2_, with Δ*T*_2_ of −5.2, in agreement with the short term pharmacokinetic, meaning that bi-magnetic NPs are slowly taken up of the by the liver. At 24 h post administration, the liver showed partial recovery in *T*_2_ values, Δ*T*_2_ of −3.1, which could be explained by hepatic excretion or by degradation the NPs and metabolization of the iron in the Kupffer cells. Additionally, in agreement with the short term pharmacokinetics, the kidneys did not show any significant variation at any time point of the study. The bladder did not show any significant *T*_2_ variation (data not shown), involving that NPs were not excreted by the kidneys, as expected for their size, which is above the glomerular filtration size limit. 

**Figure 9:**
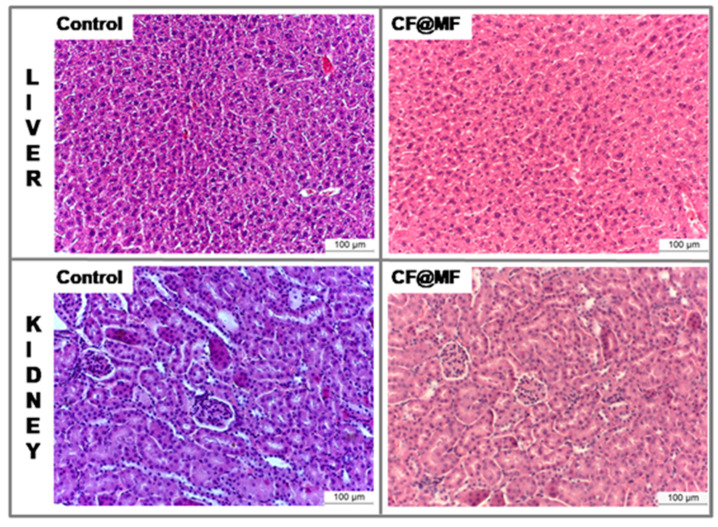
Haematoxylin and eosin representative histological sections of control liver (**a**), liver at 24 h post-injection of PEGylated CF@MF NPs (**b**), control kidney (**c**), and kidney at 24 h post-injection of PEGylated CF@MF NPs (**d**).

Finally, the possible damage of the main organs after intravenous injection of PEGylated CF@MF NPs was evaluated by histological analysis. Liver sections of H&E staining did not show any of the typical signs of acute or subacute tissue injury, such as the vacuolated swelling of the cytoplasm of hepatocytes. Similarly, the kidneys presented normal tubular brush borders and intact glomeruli surrounding the Bowman’s capsule, evidencing the absence of kidney injury (Figure 9) [45].

**Figure 10:**
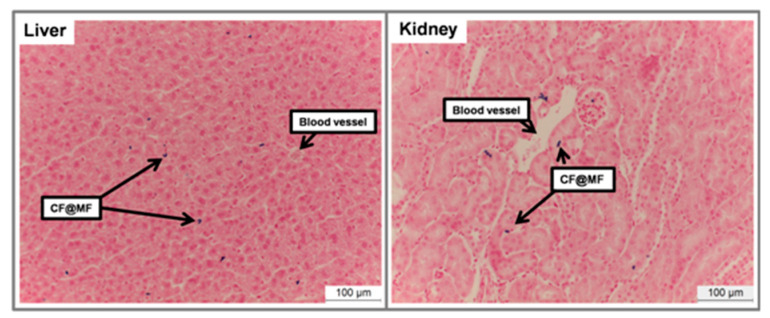
Prussian blue staining of representative histological sections at 24 h post-injection of PEGylated CF@MF NPs of liver (left) and kidney (right).

Furthermore, the accumulation of colloidal iron from NPs was evaluated by Prussian blue staining in the liver and kidneys. As can be observed in Figure 10, a homogeneous accumulation of PEGylated CF@MF NPs was observed in the liver, although at very low concentration, which is in agreement with the small *T*_2_ decrease that was observed in the MRI quantitative analysis. As for the kidneys, only a reduced presence of NPs in the areas close to the large blood vessels could be observed, which was in agreement with the almost undetectable *T*_2_ changes that were observed in the MRI studies (Figure 10).

## 4. Conclusions

In this work, we carried out the synthesis of monodisperse bi-magnetic NPs with high SAR and high magnetic relaxivity, as multifunctional biomedical nanosystems for magnetic hyperthermia and magnetic resonance imaging. The SAR values were tuned by decreasing the hard/soft volume ratio between the magnetic layers. In this regard, it is worth emphasizing the significant SAR improvement of the CoFe_2_O_4_@MnFe_2_O_4_ NPs due to the strong interfacial exchange coupling of the core-shell structure. Furthermore, we were able to provide CoFe_2_O_4_@MnFe_2_O_4_ NPs with high stability in physiological media by coating them with a PEGylated ligand, while following our previously reported method. MRI in vivo experiments showed that PEGylation conferred very good stealth properties to NPs, leading to the evasion of the mononuclear phagocyte system and, consequently, to longer circulation times and bioavailability. To the best of our knowledge, this is the first study reporting bi-magnetic CoFe_2_O_4_@MnFe_2_O_4_ NPs with combined capabilities for both cancer treatment via magnetic hyperthermia and cancer diagnosis by MRI. In conclusion, the bi-magnetic NPs that are reported herein display great potential as cancer theranostic nanosystems. 

## Figures and Tables

**Table 1:** Structural and magnetic parameters. <*d*>: mean crystallite size; a: lattice parameter; ε: microstrain. Numbers in parentheses indicate the estimated standard deviations of the last significant digit.

Sample	<*d*> (nm)	a (Å)	*ε*
CF	9.8(7)	8.3832(4)	6.55 × 10^−5^
CF@MF	10.4(8)	8.3961(2)	7.16 × 10^−4^

## Supplementary Materials

The following are available online at https://www.mdpi.com/2079-4991/10/5/907/s1, Figure S1: XRD patterns of the CF@ZF sample, Figure S2: TEM images of CF@ZF nanoparticles. The scale bar is equivalent to 20 nm in all the images. The size distribution histogram is shown on the right. Diameter is expressed as the mean ± SD, by measuring at least 100 particles, Figure S3: (a) Magnetization curves of CF@ZF nanoparticles. (b) SAR for different power values of the CF@ZF nanoparticles (at a frequency of 1950 kHz), Figure S4: FTIR spectra of the gallol-PEG-OH ligand and the functionalized magnetic nanoparticles, Table S1: Structural and magnetic parameters calculated for the CF@ZF nanoparticles. <d>: mean crystallite size of particles; a: lattice parameter; ε: microstrain. Numbers in parentheses indicate the estimated standard deviations of the last significant digit. Methods, XRD, TEM, magnetic characterization, FTIR, in vitro relaxivities, and in vitro cytotoxicity.
