# Supplementary material for: Bi-Magnetic Core-Shell CoFe2O4@MnFe2O4 Nanoparticles for In Vivo Theranostics
Source: Nanomaterials (Basel). 2020 May 8;10(5):907. doi: 10.3390/nano10050907 (PMC7279505; doi:10.3390/nano10050907)
Supplement: Supplementary file 1 [file nanomaterials-10-00907-s001.pdf]

Bi-magnetic core-shell $CoFe_2O_4@MnFe_2O_4$ nanoparticles for in vivo theranostics

Valentin Nica[1,2†], Carlos Caro[1†], Jose Maria Páez-Muñoz[1], Manuel Pernia-Leal[3*] and Maria Luisa Garcia-Martin[1,4*]

[1]BIONAND, Centro Andaluz de Nanomedicina y Biotecnología, Junta de Andalucía, Universidad de Málaga, Málaga, Spain

[2]Department of Physics, "Alexandru Ioan Cuza" University of Iasi, 700506Iasi, Romania

[3]Departamento de Química Orgánica y Farmacéutica, Universidad de Sevilla, 41012 Seville, Spain

[4]Networking Research Center on Bioengineering, Biomaterials and Nanomedicine, CIBER-BBN, Malaga, Spain

[†] Valentin Nica and Carlos Caro contributed equally to this work.

* email: mpernia@us.es; mlgarcia@bionand.es

Index

1. Methods

A) *In vitro* longitudinal and transversal relaxivities (r_1 and r_2)

B) Cytotoxicity assays

C) Histology

2. Results

A) XRD

B) TEM

C) Magnetic characterization

D) FTIR

E) *In vitro* relaxivities

F) *In vitro* cytotoxicity

G) Histology

1. Methods

A) *In vitro* longitudinal and transversal relaxivities (r1 and r2)

Regions of interest (ROIs) were drawn on the first image of the image sequence and the intensity values extracted and fit to the following equations:

$$M_Z(t) = M_0(1 - e^{-TR/T_1})$$

$$M_{XY}(t) = M_0 e^{-TE/T_2}$$

Where M_z and M_{xy} are the signal intensities at time TR or TE, and M_0 is the signal intensity at equilibrium.

B) Cytotoxicity assays

C6 cells were plated at a density of 1 x 10^4 cells/well in a 96-well plate at 37 ºC in 5 % CO_2 atmosphere (200 μL per well, number of repetitions = 5). After 24 h of culture, the medium in the wells was replaced with fresh medium containing PEGylated CF@MF gallol derived magnetic nanoparticles in varying concentrations, from 0.1 μg/mL to 100 μg/mL. After 24 h, the supernatant of each well was replaced by 200 μL of fresh medium with 3-[4,5-dimethylthiazol-2-yl]-2,5-diphenyl tetrazolium bromide (MTT) (0.5 mg/mL). After 2 h of incubation at 37 ºC and 5 % CO_2 the medium was removed and the formazan crystals were solubilized with 200 μL of DMSO, and the solution was vigorously mixed to dissolve the reacted dye. The absorbance of each well was read on a microplate reader (Dynatech MR7000 instruments) at 550 nm. The relative cell viability (%) and its error related to control wells containing cell culture medium without nanoparticles were calculated by the equations:

$$RCV(\%) = \left(\frac{[Abs]test - [Abs]Pos.Ctrl}{[Abs]Neg.Ctrl - [Abs]Pos.Ctrl}\right) \times 100$$

$$Error(\%) = \mathrm{RCVtest} \times \sqrt{\left(\frac{[\sigma]\mathrm{test}}{[\mathrm{Abs}]\mathrm{test}}\right)^2 + \left(\frac{[\sigma]\mathrm{control}}{[\mathrm{Abs}]\mathrm{control}}\right)^2}$$

where σ is the standard deviation.

C) Histology

Haematoxylin and Eosin (H&E): paraffin-embedded samples were sectioned at 7 µm thickness, then deparaffinized, rehydrated and stained with H&E, and then dehydrated in ascending concentrations of ethanol, cleared in xylene, and mounted on commercial glass slides.

Prussian Blue (PB): paraffin-embedded samples were sectioned at 7 µm thickness,then deparaffinized, rehydrated, submerged in 20% hydrochloric acid and 10% potassium ferrocyanide, washed with water and counterstained with Nuclear Fast Red, dehydrated in ascending concentrations of ethanol, cleared in xylene and mounted in commercial glass slide.

2. Results

A) XRD

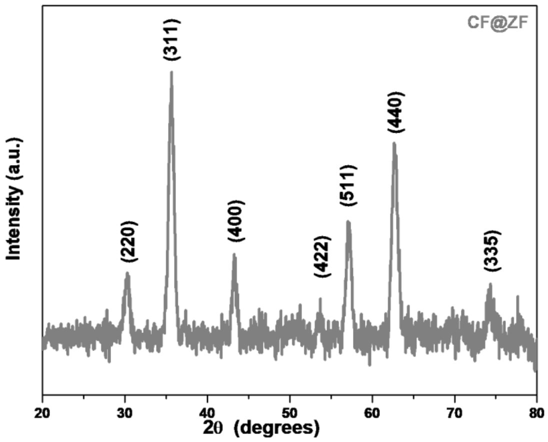

Figure S1. XRD patterns of the CF@ZF sample.

Sample	<d> (nm)	a (Å)	ε
CF@ZF	12.7(5)	8.3845(6)	2.29E-05

TABLE S1. Structural and magnetic parameters calculated for the CF@ZF nanoparticles. <d>: mean crystallite size of particles; a: lattice parameter; ε: microstrain. Numbers in parentheses indicate the estimated standard deviations of the last significant digit.

B) TEM

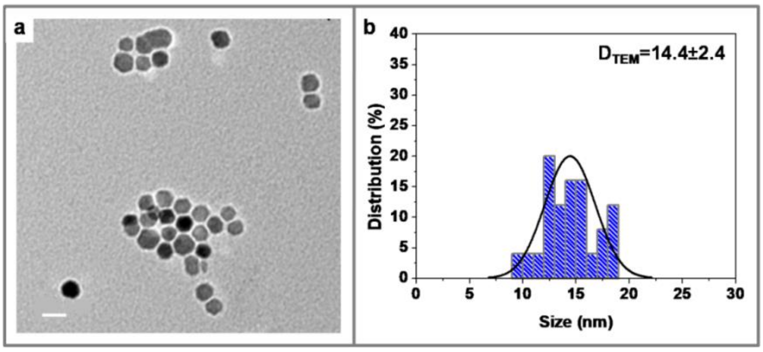

Figure S2. TEM images of CF@ZF nanoparticles. The scale bar is equivalent to 20 nm in all the images. The size distribution histogram is shown on the right. Diameter is expressed as the mean ± SD, by measuring at least 100 particles.

C) Magnetic characterization

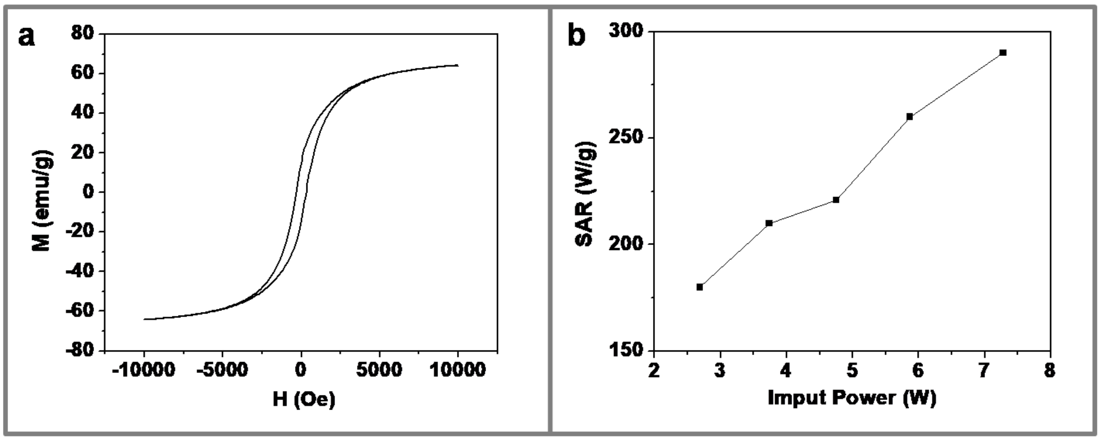

Figure S3. (a) Magnetization curves of CF@ZF nanoparticles. (b) SAR for different power values of the CF@ZF nanoparticles (at a frequency of 1950 kHz).

D) FTIR

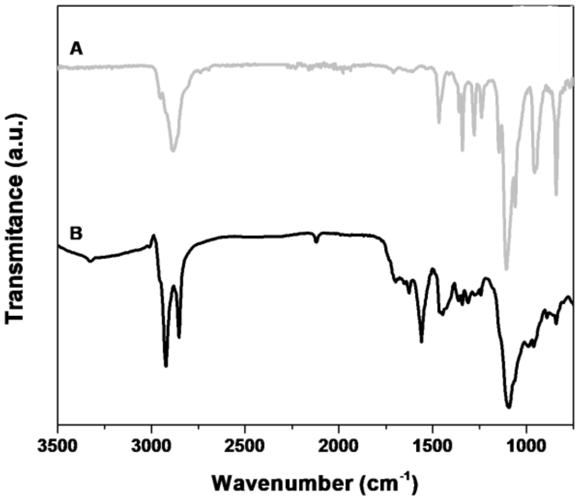

Figure S4. FTIR spectra of the gallol-PEG-OH ligand and the functionalized magnetic nanoparticles.

E) *In vitro* relaxivities

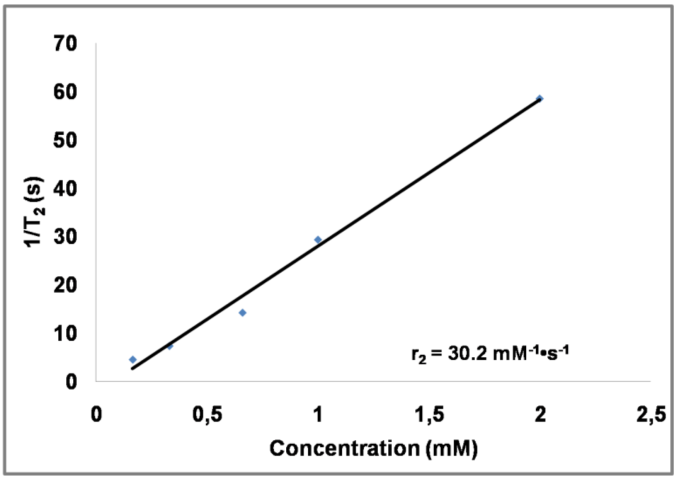

Figure S5. Plot of transverse relaxation rate ($1/T_2$) over the Fe concentration of magnetic nanoparticles, measured at 1.44 T. Transverse relaxivity (r_2) is calculated from the slope of the linear regression (black line).

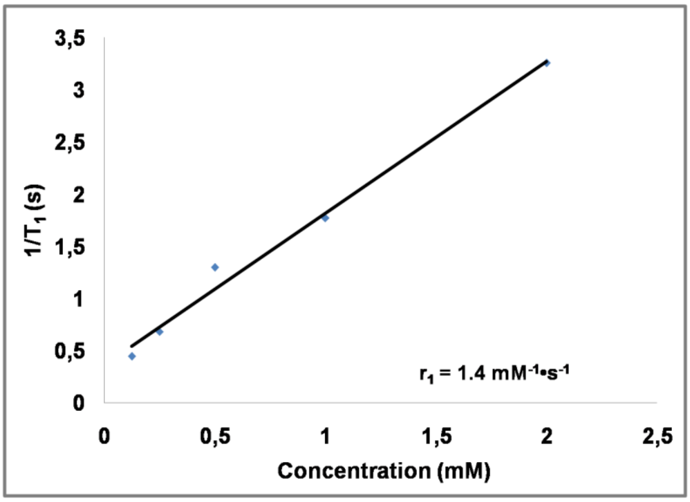

Figure S6. Plot of longitudinal relaxation rate ($1/T_1$) over the Fe concentration of magnetic nanoparticles, measured at 1.44 T. Longitudinal relaxivity (r_1) is calculated from the slope of the linear regression (black line).

F) *In vitro* cytotoxicity

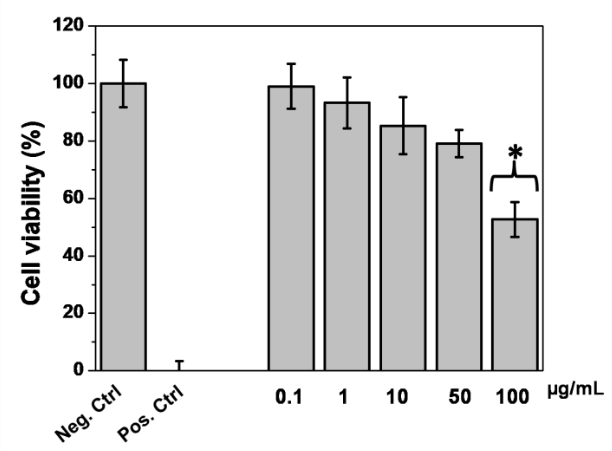

Figure S7. MTT cytotoxicity assay of magnetic nanoparticles on C6 cells treated for 24 h.

G) Histology

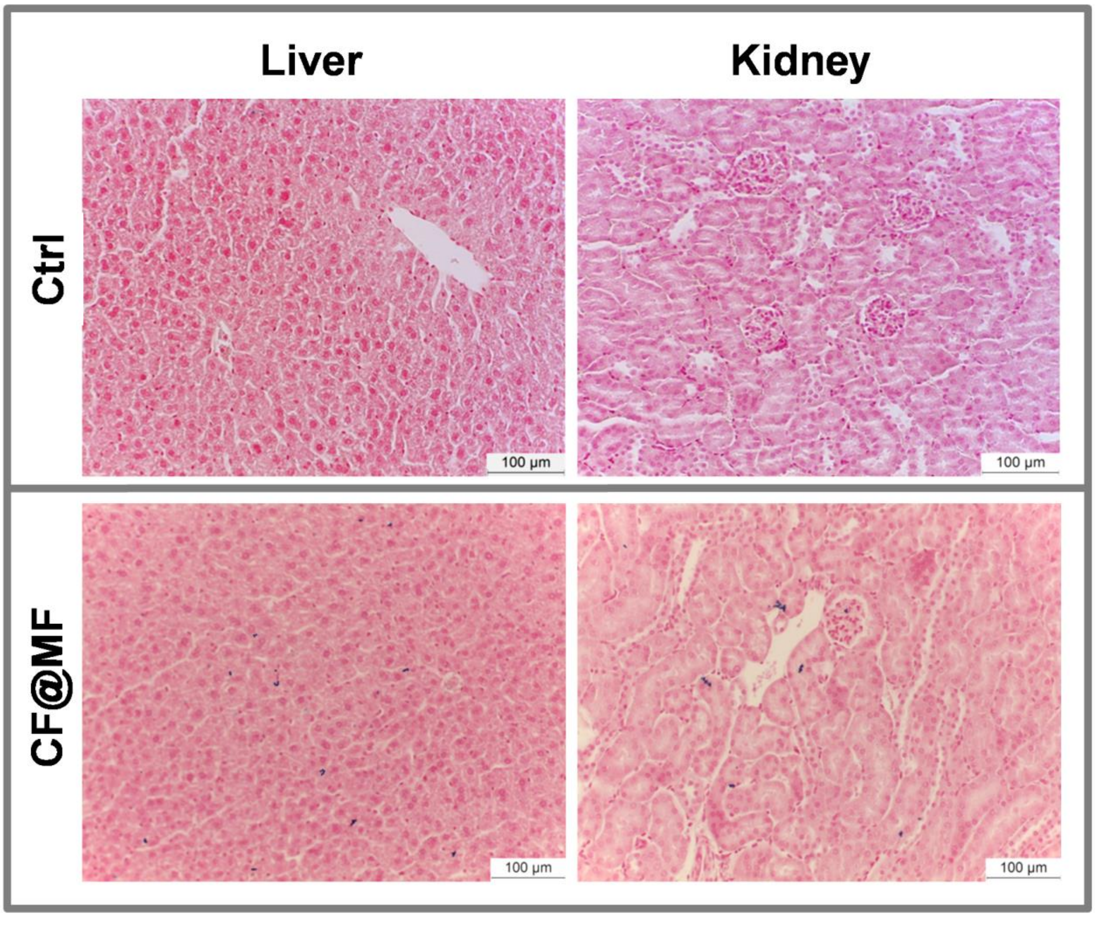

Figure S8. Prussian blue staining of representative histological sections of liver (left) and kidneys (right) after 24 h of intravenous injection of PBS (top) and PEGylated CF@MF nanoparticles (bottom).
